# Fructan-Enriched Diet Increases Bone Quality in Female Growing Rats at Calcium Deficiency

**DOI:** 10.1007/s11130-018-0671-4

**Published:** 2018-05-10

**Authors:** Kinga Topolska, Radosław Piotr Radzki, Agnieszka Filipiak-Florkiewicz, Adam Florkiewicz, Teresa Leszczyńska, Ewa Cieślik

**Affiliations:** 1 Department of Nutrition Technology and Consumption, Faculty of Food Technology, University of Agriculture in Krakow, Krakow, Poland; 2 Department of Animal Physiology, Faculty of Veterinary Medicine, University of Life Sciences in Lublin, Lublin, Poland; 3 Department of Food Analysis and Quality Assessment, Faculty of Food Technology, University of Agriculture in Krakow, Krakow, Poland; 4 Department of Human Nutrition, Faculty of Food Technology, University of Agriculture in Krakow, Krakow, Poland

**Keywords:** Fructans, Calcium deficiency, Rats, Femur, pQCT

## Abstract

This study was designed to determine the effect of feeding female rats with low-calcium diet containing one of three fructan sources (Jerusalem artichoke, yacon, Beneo Orafti Synergy1), on selected bone parameters. Growing Wistar rats were fed modified AIN-93 G diet enriched in fructan sources (8%), added alone or as a strawberry sorbet ingredient. Two of eight groups were a validation model, where the positive control group was fed with recommended calcium dose in the diet (RCD), and negative one – with low calcium diet (LCD). After 12 weeks, femoral Ca content, bone densitometry, architecture and hardness were examined. The positive effects on femoral Ca content and cortical thickness, area and content in distal part of bone was observed after feeding animals diet enriched in Jerusalem artichoke sorbet. Beneficial action on other bone tomographic parameters (particularly trabecular volumetric bone mineral density) in this part of femur were associated mainly with the consumption of the diet with sorbet containing yacon. Our results showed an important role of diet containing frozen strawberry desserts enriched in fructan sources in the maintenance of healthy bones of growing organism. It may suggest possible synergisms between fructans and bioactive substances of strawberry.

**Electronic supplementary material:**

The online version of this article (10.1007/s11130-018-0671-4) contains supplementary material, which is available to authorized users.

## Introduction

Osteoporosis is a serious, highly prevalent global health problem, being a multifactorial bone disease [1, 2]. The total health burden osteoporosis was estimated at 1,180,000 lost quality adjusted life years for the EU, mainly as a consequence of prior fractures [3]. It is suggested maximizing peak bone mass during adolescence may be the key to postponing and even preventing bone fractures due to osteoporosis later in life. A key way to accomplish this is through nutrition, mainly adequate calcium intake [2, 4]. An adequate calcium, vitamin D, and protein intake results in decreased bone remodeling, better calcium retention, decreased age-related bone loss [5, 6]. Nonetheless, dietary intakes of this mineral are below recommended levels in many countries, with consequences for bone health. Ca plays a key role in the mineralization of skeleton, and provides skeletal strength [6]. The homeostasis of this mineral refers to the hormonal regulation of Ca^2+^ in serum by parathyroid hormone, 1,25-dihydroxyvitamin D, and serum ionized calcium itself; they together regulate transport of Ca at the gut, kidney, and bone [6]. Calcium absorption is a function of active transport that is controlled by 1,25(OH)_2_D (particularly important at low Ca intakes), and passive diffusion (dominates at high Ca intakes) [6]. A sufficient intake of Ca and vitamin D can reduce the risk of fractures [1]. Many ways for improving bone health by foods are discussed [2, 7]. Highly fermentable fibres, such as inulin and fructooligosaccharides promote absorption of minerals in the colon [8]. Fructans are natural components of several fruits and vegetables [9, 10]. Among significant sources of these compounds, Jerusalem artichoke (*Helianthus tuberosus* L.), yacon (*Smallanthus sonchifolius*) and chickory (*Cichorium intybus*) are to be mentioned. Inulin-type fructans are classified as functional food ingredients [9]. Through their gastrointestinal effects, they may indirectly affect the immunological function and the metabolism of carbohydrates, lipids and minerals. The positive effects of inulin-type fructans on mineral absorption and architecture of bone were described by several authors [2, 4, 11]. Fructans may vary with their potential to stimulate mineral absorption or accretion in the skeleton. This effect may depend on the degree of polymerization (DP); it is assumed that oligofructose (with shorter DP) will be fermented more rapidly in the upper large intestine, whereas inulin will be fermented predominantly in the lower part. A mixture of short-chain and long-chain inulin will be fermented all along the whole gut, and thus, more effectively [12]. Fructans are used to improve structure and/or taste of food products. They have a low energy value, are safe, and generally well tolerated up to a level of 20 g/d [13]. Although health benefits of fructans have been well established, the effect of their consumption (fructan enriched sorbets) on the organism are not characterized yet. The concept for choosing this kind of product was in the line with the recommendation of WHO to increase daily intake of vegetables and fruits [14]. Strawberry is a rich source of vitamins, fiber, and phytochemicals [15, 16]. It is a very popular raw material in food industry (*e.g.* jam, ice creams). Frozen desserts can be used to carry health-promoting constituents – vitamins, minerals, fiber, etc. [17]. This study is unique in that it compares various fructan sources and forms of addition, in the same model (growing female rat). The major question is, whether examined fructan sources (alone or added to the strawberry sorbets) could act beneficially on bone quality, under condition of dietary calcium deficiency.

## Materials and Methods

### Materials

Research materials were: the pulp from tubers of Jerusalem artichoke (*Helianthus tuberosus* L.) var. Albik (Agricultural holding using sustainable system of plant production, Wielkopolska Region, Poland), yacon root (*Smallanthus sonchifoulis*) powder (from Oxapampa, Peru - EverTrust, United Kingdom) and Beneo Orafti Synergy 1 (Belgium) used as the ingredients of strawberry sorbets. For the sorbet production, strawberries (*Fragaria* × *ananassa* Duchesne) var. Senga Sengana (Oerlemans Foods, Poland) were used. The recipe and preparation of sorbets was described in detail elsewhere [18]. These products contained calcium in the amount 7.82 mg/100 g (sorbet containing Jerusalem artichoke), 15.09 mg/100 g (sorbet containing yacon root powder), and 4.05 mg/100 g (sorbet containing Beneo Orafti Synergy 1).

### Animals Diets and Experimental Design

Sixty-four female Wistar rats with mean body weight of 126 g were housed in a controlled environment (20-23 °C, 12 h/12 h day:night) in common cages. After seven days of acclimatization, the animals were randomly divided into experimental groups as follows: validation model, with two groups (*n* = 8/group); group I - rats fed modified AIN-93G diet [19] with recommended calcium dose-5000 mg/kg diet (RCD) and group II - rats fed a low calcium (40% of Ca deficiency) diet (LCD); treatment, with six groups (*n* = 8/group); rats fed low-calcium diet containing fructan sources (in the amount providing 8% fructans in diet), *i.e.*: Jerusalem artichoke pulp, yacon root powder and the formulation Beneo Orafti Synergy 1, all added alone (LCD-JA, LCD-Y, LCD-F) or in the sorbet (LCD-JAS, LCD-YS, LCD-FS), respectively. The Ca content in LCD groups was on the level of 3000 ± 0.1 mg/kg diet. Jerusalem artichoke pulp as well as all sorbets were added to the diet after lyophilization (Christ Alpha 1–4 apparatus, Germany). Institutional and national guidelines for the care and use of laboratory animals were followed. All the procedures were approved by First Local Ethical Committee on Animal Testing at the Jagiellonian University in Krakow (no 119/2011). The formulations of treatment diets (containing all fructan sources) were calculated to provide equal contents of calcium and fiber, including fructan amount (Table 1 – Supplementary Material). The rats were allowed free access to the food and deionised water throughout the experiment period (12 weeks). The weight gain and the consumption of diet were monitored and no significant differences (*p* > 0.05) were noted. At the end of the experiment rats were euthanized (decapitation), according to the ethical guidelines for animal experimentation.

### Femora Sampling and Analyses

The rats’ hind legs were dissected. To prevent desiccation of the bone samples, the legs were frozen at -80 °C until measurements. The femora were then dissected, and next separated with blade surgery, eliminating the tissue adhering to the bone. The femoral calcium content was measured according to CSN EN 15505 [20], by AAS method with flame atomization (Varian AA240FS, USA). Prior to analysis, wet mineralization (maximum temperature set at 200 °C, mineralization time: 40 min) was performed using MarsXpress (CEM, USA), with 10 ml 65% nitric acid (Suprapur® Merck, Germany). The accuracy of methods used was verified on the basis of certified reference material NCS ZC73012—GSB-5. All the methods used are fully validated, and checked by internal quality control procedure [21] and interlaboratory/proficiency tests. The analysis of bone hardness was performed by the use of texture analyzer TA-XT Plus (Stable Micro System LTD, Godalming, Surrey, UK) with a Warner-Bratzler attachment consisted of 3 mm thick steel blade (73°V cut into its lower edge fitted through a 4 m wide slit in platform). The parameters of analysis were: pre-test speed 2.0 mm/s; test-speed 5.0 mm/s; post-test speed 10 mm/s; trigger force 10 G; distance 10 mm. Bones were scanned with peripheral quantitative computed tomography (pQCT) XCT Research SA Plus system with software version 6.2 C (Stratec Medizintechnik GmbH, Pforzheim, Germany). The scans were performed in the distal metaphysis (5 mm from distal end) for the analysis of the trabecular bone tissue and also in the middle diaphysis (50% of bone length) for the analysis of the cortical bone tissue. The following parameters were determined: cortical thickness (DIS-CRT_THK, MID-CRT_THK), cortical area (DIS-CRT_A, MID-CRT_A), cortical density (DIS-CRT_DEN, MID-CRT_DEN), cortical content (DIS-CRT_CNT, MID-CRT_CNT), trabecular area (DIS-TRAB_A, MID-TRAB_A), total area (DIS-TOT_A, MID-TOT_A), trabecular density (DIS-TRAB_DEN, MID-TRAB_DEN), trabecular content (DIS-TRAB_CNT, MID-TRAB_CNT), total density (MID-TOT_DEN), total content (MID- TOT_CNT), polar strain index (RP-CM-W), as well as periosteal (DIS-PERI_C MID-PERI_C) and endosteal (DIS-ENDO_C, MID-ENDO_C) circumferences. The cortical tissue was tested with a threshold of 0.900 cm^−1^ and cortical mode 2. In turn, analyses of the trabecular bone were performed with a threshold of 0.450 cm^−1^, contour mode 2, and a peel mode 20. The initial scan was performed at a speed of 10 mm/s, and CT-scan 4 m/s. The pQCT system was calibrated with the use of hydroxyapatite, containing a quality assurance phantom (pQCT QA-Phantom).

### Statistical Analysis

Data were presented as mean value ± SD. Differences between RCD and LCD groups were analyzed by the Student’s *t*-test for unpaired samples. Results from treatment groups were subjected to two-way analysis of variance, where the factor 1 was the kind of fructan source, factor 2 - form of added fructan-containing source (alone or in the sorbet). A *post hoc* Duncan’s test was used. Differences were considered significant at *p* < 0.05. Hypothesis about normal distribution was verified by Shapiro-Wilk test, and homogeneity of variance - by Levene’a test. For a better illustration of differences between experimental groups, principal component analysis (PCA) was also done in two versions; the first one included all experimental groups, while the second one – only groups fed low-calcium diets (without RCD group). All calculations were performed with statistical software package Statistica 9.1 (StatSoft Inc., USA).

## Results and Discussion

Nutrition plays a crucial role in bone stock. When Ca^2+^ level in serum falls due to low calcium intake, parathormone release rises, provoking bone resorption stronger than building-up [22]. As it was widely reviewed by Morris et al. [23], animal models fed low Ca diets demonstrate a negative calcium balance and bone loss. The disturbance in Ca homeostasis induced by its dietary deficiency was also observed in our study in terms of femoral quality.

### Calcium Content in Femur

Tabular summaries of the changes between group means of relevant bone quality parameters are available in Table 1. When analyzing the results concerned on calcium bone status in validation model, statistically significant decrease in the content of this mineral among animals from LCD group compared to RCD animals was observed. In the study of Hong et al. [24], dietary Ca deficiency in growing rats after eight weeks also led to significantly decreased mineral content.

**Table 1:** Effects of fructan-enriched diet different on selected parameters of bone condition in rats (mean value ± SD)

Parameter (unit)	Validation model		Treatments					
			Kind of fructan source (raw material)			Kind of sorbet (in dependence on fructan source)		
	RCD	LCD	LCD-JA	LCD-Y	LCD-F	LCD-JAS	LCD-YS	LCD-FS
Femoral Ca (mg/g)	153.39^a^ ± 8.73	140.14^bB^ ± 4.40	138.20^AB^ ± 10.27	126.19^A^ ± 12.59	145.24^B^ ± 7.22	202.89^D^ ± 17.20	133.32^AB^ ± 14.27	158.27^C^ ± 9.90
Hardness (N)	122.54 ± 18.83	119.78 ± 17.31	150.77 ± 33.78	138.62 ± 12.87	125.89 ± 6.21	147.73 ± 25.91	133.86 ± 18.42	129.23 ± 17.42
DIS-ENDO_C (mm)	12.29 ± 0.66	12.18 ± 0.87	11.79 ± 0.70	11.97 ± 0.61	12.16 ± 0,53	11.64 ± 0.50	11.78 ± 0.70	11.95 ± 0.96
DIS-PERI_C (mm)	15.06 ± 0.67	14.83 ± 0.78	14.61 ± 0.69	15,01 ± 0,48	14,64 ± 0,29	15.05 ± 0.90	15,11 ± 0,64	14,99 ± 0,71
DIS-CRT_THK(mm)	0.44 ± 0.06	0.42^AB^ ± 0.07	0.45^AB^ ± 0.09	0.48^AB^ ± 0.10	0.40^A^ ± 0.05	0.54^B^ ± 0.10	0.53^AB^ ± 0.09	0.48^AB^ ± 0.11
DIS-CRT_A (mm^2^)	6.03 ± 0.82	5.67^AB^ ± 0.99	5.94^AB^ ± 1.24	6.51^AB^ ± 1.34	5.28^A^ ± 0.62	7.28^B^ ± 1.59	7.11^AB^ ± 1.24	6.48^AB^ ± 1.35
DIS-CRT_DEN (mg/mm^3^)	1189.47 ± 37.65	1223.37 ± 27.61	1213.16 ± 38.26	1193.96 ± 29.01	1219.08 ± 34.29	1187.07 ± 38.27	1167.45 ± 34.14	1194.01 ± 50.21
DIS-CRT_CNT (mg/mm)	7.15 ± 0.79	6.93^AB^ ± 1.15	7.18 ^AB^ ± 1.33	7.74^AB^ ± 1.44	6.45^A^ ± 0.86	8.60^B^ ± 1.67	8.27^AB^ ± 1.28	7.71^AB^ ± 1.48
DIS-TRAB_A (mm^2^)	8.12 ± 0.72	7.89 ± 0.82	7.66 ± 0.71	8.07 ± 0.52	7.68 ± 0.31	8.14 ± 0.94	8.18 ± 0.69	8.06 ± 0.78
DIS-TOT_A (mm^2^)	18.07 ± 1.59	17.54 ± 1.83	17.03 ± 1.60	17.94 ± 1.16	17.07 ± 0.68	18.09 ± 2.10	18.19 ± 1.53	17.91 ± 1.73
DIS-TRAB_DEN (mg/mm^3^)	559.21^a^ ± 90.15	438.84^bA^ ± 77.04	568.90^AB^ ± 88.04	562.50^AB^ ± 94.20	457.89^A^ ± 53.00	565.20^AB^ ± 137.86	643.16^B^ ± 54.62	553.59^AB^ ± 104.78
DIS-TRAB_CNT (mg/mm)	4.56^a^ ± 0.93	3.50^bA^ ± 0.92	4.41^AB^ ± 1.06	4.57^AB^ ± 1.00	3.52^A^ ± 0.45	4.71^AB^ ± 1.53	5.28^B^ ± 0.78	4.50^AB^ ± 1.12
DIS-TOT_DEN (mg/mm^3^)	807.74 ± 45.97	758.16^A^ ± 39.36	826.30^AB^ ± 48.51	821.68^AB^ ± 56.49	760.66^A^ ± 26.17	835.94^AB^ ± 71.44	862.59^B^ ± 34.22	822.05^AB^ ± 63.03
DIS-TOT CNT (mg/mm)	14.61 ± 1.62	13.31 ± 1.74	14.11 ± 1.93	14.77 ± 1.73	12.98 ± 0.53	15.24 ± 2.84	15.69 ± 1.52	14.73 ± 1.89
MID-ENDO_C (mm)	5.91 ± 0.57	6.03 ± 0.46	6.05 ± 0.37	5.87 ± 0.35	6.12 ± 0.23	6.16 ± 0.36	6.00 ± 0.30	6.01 ± 0.50
MID-PERI_C (mm)	10.01 ± 0.55	10.21 ± 0.53	10.10 ± 0.44	9.99 ± 0.48	10.13 ± 0.36	10.28 ± 0.50	10.05 ± 0.27	10.09 ± 0.47
MID-CRT_THK(mm)	0.65 ± 0.03	0.67 ± 0.03	0.64 ± 0.02	0.66 ± 0.03	0.64 ± 0.03	0.66 ± 0.03	0.64 ± 0.02	0.65 ± 0.03
MID-CRT_A (mm^2^)	5.19 ± 0.40	5.42 ± 0.47	5.21 ± 0.38	5.21 ± 0.45	5.19 ± 0.40	5.41 ± 0.49	5.17 ± 0.21	5.23 ± 0.39
MID-CRT_DEN (mg/mm^3^)	1436.86 ± 9.20	1436.42 ± 14.21	1440.55 ± 16.38	1450.16 ± 4.23	1439.55 ± 9.45	1445.00 ± 11.21	1441.35 ± 10.73	1443.36 ± 16.07
MID-CRT_CNT (mg/mm)	7.45 ± 0.54	7.78 ± 0.63	7.51 ± 0.53	7.55 ± 0.65	7.46 ± 0.59	7.82 ± 0.70	7.45 ± 0.28	7.55 ± 0.55
MID-TRAB_A(mm^2^)	3.60 ± 0.40	3.75 ± 0.40	3.66 ± 0.31	3.58 ± 0.34	3.68 ± 0.26	3.80 ± 0.36	3.62 ± 0.20	3.65 ± 0.34
MID-TOT_A (mm^2^)	7.99 ± 0.88	8.08 ± 0.55	8.13 ± 0.70	7.96 ± 0.75	8.17 ± 0.58	8.44 ± 0.80	8.05 ± 0.44	8.12 ± 0.76
MID-TRAB_DEN (mg/mm^3^)	470.24 ± 68.49	449.95 ± 74.16	435.70 ± 63.96	467.94 ± 52.31	384.64 ± 65.24	423.17 ± 56.19	444.45 ± 60.16	408.10 ± 89.52
MID-TRAB_CNT (mg/mm)	1.67 ± 0.16	1.67 ± 0.24	1.59 ± 0.19	1.67 ± 0.17	1.42 ± 0.27	1.60 ± 0.21	1.61 ± 0.21	1.42 ± 0.27
MID-TOT_DEN (mg/mm^3^)	1000.61 ± 37.53	993.42 ± 40.59	988.26 ± 33.80	1014.63 ± 23.49	968.93 ± 25.74	986.29 ± 29.40	993.55 ± 26.92	982.01 ± 48.44
MID- TOT_CNT (mg/mm)	7.98 ± 0.65	8.25 ± 0.69	8.03 ± 0.60	8.07 ± 0.69	7.92 ± 0.62	8.31 ± 0.73	7.99 ± 0.39	7.95 ± 0.62
RP-CM-W (mm^3^)	5.46 ± 0.86	5.77 ± 0.77	5.54 ± 0.67	5.47 ± 0.68	5.60 ± 0.55	5.92 ± 0.79	5.42 ± 0.37	5.69 ± 0.64

a, b - unlike small letters mean significant differences between RCD and LCD; A, B - unlike capital letters mean significant differences among LCD groups

RCD - diet with recommended calcium dose; LCD – calcium deficient diet (60% recommended Ca dose), LCD-JA: low-calcium diet enriched in Jerusalem artichoke, LCD-Y: low-calcium diet enriched in yacon, LCD-F: low-calcium diet enriched in Beneo Orafti Synergy 1, LCD-JAS: low-calcium diet enriched in sorbet containing Jerusalem artichoke, LCD-YS: low-calcium diet enriched in sorbet containing yacon, LCD-FS: low-calcium diet enriched in sorbet containing Beneo Orafti Synergy 1 cortical thickness (DIS-CRT_THK, MID-CRT_THK), cortical area (DIS-CRT_A, MID-CRT_A), cortical density (DIS-CRT_DEN, MID-CRT_DEN),cortical content (DIS-CRT_CNT, MID-CRT_CNT), trabecular area (DIS-TRAB_A, MID-TRAB_A), total area (DIS-TOT_A, MID-TOT_A), trabecular density (DIS-TRAB_DEN, MID-TRAB_DEN), trabecular content (DIS-TRAB_CNT, MID-TRAB_CNT), total density (MID-TOT_DEN), total content (MID- TOT_CNT), polar strain index (RP-CM-W), as well as periosteal (DIS-PERI_C MID-PERI_C) and endosteal circumferences (DIS-ENDO_C, MID-ENDO_C) where DIS – means distal part of bone; MID – means middle part of bone

Two-factorial analysis of variance confirmed a significant impact not only fructan source (factor 1) and form of their addition (factor 2) but also interaction between them (factor 1 x factor 2) on femoral Ca level (Table 2 - Supplementary Material). The consumption of LCD-JAS diet as well as LCD-FS diet led to enhancement (*p* < 0.05) of Ca amount in rat femur, compared with LCD animals. Simultaneously, LCD-Y animals had significantly lower femoral calcium content in comparison to LCD, LCD-F, LCD-JAS and LCD-FS (Table 1). On the contrary, Lobo et al. [11] showed that yacon flour (7.5% FOS) in the diet caused a significant increase in Ca concentration in femur of growing rats. The reason for the observed differences could be the research material characteristic (especially fructan DP). The content of fructans as well as the polymerization degree in plant material is diversified because of climatic conditions during vegetation and harvest time [18].

It is to emphasize that the increments in mineral retention, even if they are small, may contribute to a more stable trabecular network or locally higher mineralization of bone with large effects on bone stability [25]. The increase in the femoral Ca content correlates strongly with the increase of calcium absorption with several underlying mechanisms [26]. The effects of fructans on Ca absorption appear to be modulated by genetic factors, including genetic polymorphism of a specific D-vitamin receptor. It has been observed that the consumption of inulin-type fructans increases Ca bioavailability and, in turn, enhances bone density and mineral mass [9]. However, the effect of dietary fructans on calcium bioavailability and bone density may vary with their degree of polymerization [26]. It is assumed that oligofructose (with shorter DP) will be fermented more rapidly in the upper large intestine, whereas inulin (longer DP) will be fermented predominantly in the lower part, and a mixture of short-chain and long-chain inulin (Synergy) will be fermented all along the whole gut and thus more effectively [12]. The results of the study of Kruger et al. [4] showed that FOS with different DP have different effects on calcium absorption and retention. Inulin (DP > 23) increased Ca absorption significantly compared with control, and this calcium was retained in the bone as shown by increased Ca balance. Nzeusseu et al. [27] reported that among the fructans tested (OF, high-performance inulin, Synergy1 and branched-chain inulin), only the group of animals receiving the Synergy1 presented higher intestinal Ca absorption compared to the control group. The authors suggested that this phenomenon could be explained by synergistic effects of fermentable carbohydrates with different DP on intestinal absorption of Ca.

It is to emphasize that DP of yacon fructans was between 3 and 7. Meanwhile, Jerusalem artichoke fructans are characterized by higher DP values (especially tubers harvested in autumn – 19). In the present study, the JA tubers from autumn harvest was used for sorbet production [18]. According to manufacturer information Synergy 1® is a dedicated combination of longer and shorter chain inulin to achieve specific physiological effect.

When regarding the maintenance of adequate calcium stores in bone, consuming LCD-JAS diet was the most advantageous in this aspect (44% femoral Ca content increase, as compared to LCD).

### Bone Hardness

After a 3-month period corresponding to ca. 3 bone modeling–remodeling cycles, calcium dietary deficiency caused only slightly lower hardness of femora (by 2.25% compared to RCD) (Table 1). Treatment groups were characterized by higher values (*p* > 0.05) of femoral hardness, from 5% (LCD-F) to 25% (LCD-JA), in comparison to the LCD animals. It should be kept in mind that bone strength depends not only on Ca content alone but also on the architectural properties of bone material and other mineralization unrelated factors (*i.e.*, crystal arrangement, size, distribution of tissue microdamage) [11, 28]. Two-factorial analysis of variance showed that only the kind of fructan source had a significant impact on bone hardness (Table 2 – Supplementary Material).

### Architecture of Femur

Animal studies provide models for investigating the mechanisms by which such adverse outcomes may arise allowing the investigation of bone architecture [24, 26]. The decrease (*p* < 0.05) in LCD group in DIS-TRAB_DEN (by 27.1%) and DIS-TRAB_CNT (by 23.2%) was revealed (Table 1). The other parameters in this area of bone were not statistically differentiated, but values noticed for LCD group tended to be lower than RCD (except for DIS-CRT_DEN). Hong et al. [24] showed that the consumption of Ca-deficient diet for 8 weeks led to disorder of ultrastructure at the femoral distal diaphysis in growing rats. Two-factorial analysis of variance showed that the kind of fructan source had a significant impact on DIS-TRAB_DEN, DIS-TRAB_CNT and DIS-TOT_DEN as well as MID-TRAB_DEN (Table 2 – Supplementary Material). The form of fructan source was important factor (*p* < 0.05) for DIS-CRT_THK, DIS-CRT_A, DIS-CRT_DEN, DIS-CRT-CNT, DIS-TRAB_DEN, DIS-TRAB_CNT, DIS-TOT_DEN and also DIS-TOT_CNT, while interaction between examined factors was not significant in any pQCT parameter (Table 2 – Supplementary Material). The increase of the most parameters of bone architecture in all fructan groups (in comparison to LCD group) was also observed (Table 1). Distal femoral values of trabecular density and also trabecular content were significantly increased in LCD-YS animals by 31.8 and 33.7%, when compared to LCD rats, and 28.8 and 33.3% (respectively) - versus LCD-F group. DIS-TOT_DEN was also markedly increased in LCD-YS animals in comparison with LCD and LCD-F groups. The results confirmed beneficial effect of LCD-JAS diet on cortical thickness, area and content in distal part of bone. The distal area of femora of LCD-JAS rats exhibited significantly higher values of these parameters, compared with LCD-F (Table 1). Moreover, it was observed that the impact on trabecular parameters of femur was depended on the form of fructan source. Despite of the lack of statistically significant differences, diet enriched in all sorbets had more beneficial effect on distal trabecular content (the increase by 27% for sorbet with commercial formulation, 15.5% for sorbet with yacon, and 6.8% for sorbet with Jerusalem artichoke) than each source alone. This “matrix effect” was also seen in the study of Demigné et al. [29], on a growing rat model, when the effects on mineral metabolism of diets containing 7.5% inulin or dehydrated chicory were studied. The authors observed that bone parameters were affected by the chicory diet whereas the purified inulin diets were less effective. Another example could be a study of Utami et al. [30], comparing yacon tuber with commercialized FOS in terms of physiology, fermentation products and intestinal microbial communities in rats. These authors proved that the differences in physiological and microbiological effects between the yacon tuber and FOS were more greatly affected by the yacon tuber rather than FOS. In our study, significant differences were also observed between LCD-YS and LCD-F in such parameters of the trabecular compartment established in distal femur metaphysis as distal trabecular content and distal total density, with highest values obtained for animals consuming yacon in their diet. The addition of fructans to rat diet did not impair bone circumferences of distal area, however positive tendencies were noted for animals fed diet enriched in yacon as well as all sorbets. When regarding the kind of fructans (with different DP), Coudray et al. [26] detected an increase in the area and mineral density of the cancellous bone of both the proximal tibia and vertebra in rats fed with fructans, with greater effect observed in animals fed inulin than oligofructose. In our study we did not observe significant differences in the middle area of femur; the changes observed in this area of bone were definitely milder. The same observation was made by Coudray et al. [26] and explained by higher rate of turnover in distal part of bone than in the middle one, and - in turn - higher sensitivity of trabecular than cortical compartment. However, when the middiaphysis of femur (which contains cortical bone alone), was analyzed by Coudray et al. [26], the increase in volumetric bone mineral density reached statistical significance but only in the inulin group. According to Scholz-Ahrens and Schrezenmeir [31], the beneficial effect of oligofructose on bone mineral content and trabecular structure depends on its amount in the diet, the amount of Ca in diet, duration of intervention and on the investigated skeletal site. These authors observed stimulating effects of oligofructose on Ca content and trabecular area in bone, but significant if the dietary Ca was high (1%). Coudray et al. [26] observed also increased polar stress/stain index in the inulin group (13.5%), but not in the oligofructose group (6.7%), therefore suggesting that a substantial increase in cortical bone resistance to bending might be associated with inulin consumption. Although our pQCT analysis did not show significant changes in this parameter between experimental groups, animals from LCD-JAS group had higher value of this parameter than rats from the other groups.

### Principal Component Analysis

The estimation of the impact of dietary intervention is interrelated with the evaluation of its effect on several parameters describing the organism condition. From one side, these parameters are differentiated and show a high variability. On the other hand, they are strongly associated. For better illustration of differences, the usage of appropriate method leading to the reduction of dimensions and easier interpretation of the obtained results is crucial. This is why, principal component analysis (PCA) was applied. There were two versions of PCA; the first one included all groups, and the second one - all groups except for RCD (to eliminate variability of diets and, thus, evaluate only the kind and form of fructan sources in diet). The results of PCA are presented in Fig. 1–2 (Supplementary Material). When regarding the first version as the result of the reduction of 27 variables - 7 reduced (eigenvalues 9.83, 8.06, 4.55, 1.85, 1.23, 1.05, 0.43) variables (explaining 100% variability) were obtained. Simultaneously, among calcium-deficient groups, 100% variability for 27 parameters was explained by 6 reduced variables (eigenvalues 10.46, 8.10, 4.60, 1.83, 1.39, 0.62). Such strong reduction indicates strong dependency between analyzed parameters. For the limitation of the number of data, only three first variables were considered in further analysis (both versions), explaining 83.1% (first version, Fig. 1A) and 85.79% (second version, Fig. 1B) of variability. When analyzing first version, explicit difference between values characterizing LCD and RCD groups was observed (Fig. 2A). Significant differences were noticed for all three reduced variables. In turn, analyzing the effect of fructan source, explicit difference was observed for LCD-JAS in relation to LCD and other treatment groups. Two first variables explained over 68% of variability (Fig. 2B). On the basis of our PCA results, concerned on the analysis of the share of particular real variables in reduced variables, one can conclude that the variability in bone quality of its distal part is affected not only by source of fructan, but also form of addition to the diet. Although a minor difference was showed in the case of LCD-YS and LCD-Y, the form of fructan addition to the diet was important in the case of Jerusalem artichoke and the formulation. The changes in the middle part of bone were most strongly exposed in the JAS group (variable 2,with the biggest number of MID parameters).

Fructan added to the animal diets as the component of strawberry sorbets had more beneficial effects on bone quality, in comparison to raw materials. Strawberry is a good source of vitamin C, and phenolic compounds including flavonoids and phenolic acids, such as hydroxycinnamic acids, ellagic acids, ellagitannins, xavan-3-ols, xavonols, anthocyanins, etc. The last mentioned compounds can protect bone health through reduction of oxidative stress [32]. Therefore, the reason for the more beneficial action of diet containing strawberry sorbets enriched in fructan sources could be derived from other bioactive compounds of this kind of product (*i.e.*, antioxidants), its specific physicochemical properties (*i.e.*, acidity) as well as the potential for enhancing of fructan action.

## Conclusion

We conclude that loss of bone Ca caused by the consumption of Ca-deficient diet in growing rats was prevented the most by Jerusalem artichoke sorbet. This diet exert positive action also on cortical thickness, and area as well as bone mineral content in distal metaphysis of femur. Beneficial effects on bone architecture in this part were associated mainly with the consumption of the diet with sorbet containing yacon. Both factors, *i.e.*, the kind of fructans and the form of their addition to the diet significantly affected femoral Ca content and trabecular volumetric bone mineral density, trabecular bone mineral content and total density of distal metaphysis. More advantageous effects of the diet enriched in fructan sources being a component of strawberry sorbet than added to the diet alone, on bone parameters suggest possible synergisms between fructans and bioactive substances of strawberry. Future studies are needed in this area.

## Electronic supplementary material

Supplementary Fig. 1 (DOCX 63 kb)

Supplementary Fig. 2 (DOCX 894 kb)

Supplementary Table 1 (DOCX 23 kb)

Supplementary Table 2 (DOCX 13 kb)
