# Supplementary material for: Fructan-Enriched Diet Increases Bone Quality in Female Growing Rats at Calcium Deficiency
Source: Plant Foods Hum Nutr. 2018 May 10;73(3):172–9. doi: 10.1007/s11130-018-0671-4 (PMC6096893; doi:10.1007/s11130-018-0671-4)
Supplement: Supplementary file 1 — (DOCX 63 kb) [file 11130_2018_671_MOESM1_ESM.docx]

A

**Figure 1** Principal component analysis with distribution of analyzed samples:

A - all groups, including RCD;

B - without RCD sample

(where KF - means kind of fructans and KS - means kind of sorbets)

B

RCD: diet with recommended calcium dose, LCD: low-calcium diet, LCD-JA: low-calcium diet enriched in Jerusalem artichoke, LCD-Y: low-calcium diet enriched in yacon, LCD-F: low-calcium diet enriched in Beneo Orafti Synergy 1, LCD-JAS: low-calcium diet enriched in sorbet containing Jerusalem artichoke, LCD-YS: low-calcium diet enriched in sorbet containing yacon, LCD-FS: low-calcium diet enriched in sorbet containing Beneo Orafti Synergy 1
