# Supplementary material for: Fructan-Enriched Diet Increases Bone Quality in Female Growing Rats at Calcium Deficiency
Source: Plant Foods Hum Nutr. 2018 May 10;73(3):172–9. doi: 10.1007/s11130-018-0671-4 (PMC6096893; doi:10.1007/s11130-018-0671-4)
Supplement: Supplementary file 2 — (DOCX 894 kb) [file 11130_2018_671_MOESM2_ESM.docx]

A

**Figure 2** Principal component analysis with distribution of analyzed parameters:

1. for all groups, including RCD;
2. without RCD group

RCD: diet with recommended calcium dose

B
