# Supplementary material for: Fructan-Enriched Diet Increases Bone Quality in Female Growing Rats at Calcium Deficiency
Source: Plant Foods Hum Nutr. 2018 May 10;73(3):172–9. doi: 10.1007/s11130-018-0671-4 (PMC6096893; doi:10.1007/s11130-018-0671-4)
Supplement: Supplementary file 3 — (DOCX 23 kb) [file 11130_2018_671_MOESM3_ESM.docx]

**Table 1** Composition of experimental diets

| Ingredient (g/kg) | RCD | LCD | LCD-JA | LCD-Y | LCD-F | | LCD-JAS | LCD-YS | LCD-FS |
| --- | --- | --- | --- | --- | --- | --- | --- | --- | --- |
| corn starch | 532.486 | 532.486 | 428.886 | 432.150 | 452.046 | | 146.08 | 139.06 | 131.05 |
| casein | 200.00 | 200.00 | 200.00 | 200.00 | 200.00 | | 200.00 | 200.00 | 200.00 |
| cellulose | 50.00 | 50.00 | 31.50 | 28.07 | 50.00 | | 23.05 | 19.32 | 50.00 |
| sucrose | 100.00 | 100.00 | 77.17 | 10.63 | 91.61 | | 44.07 | 0.00 | 0.00 |
| soybean oil | 70.00 | 70.00 | 70.00 | 70.00 | 70.00 | | 70.00 | 70.00 | 70.00 |
| mineral mix* | 35.00 | 35.00** | 35.00** | 35.00** | 35.00** | | 35.00** | 35.00** | 35.00** |
| vitamin mix* | 10.00 | 10.00 | 10.00 | 10.00 | 10.00 | | 10.00 | 10.00 | 10.00 |
| choline bitartate | 2.50 | 2.50 | 2.50 | 2.50 | 2.50 | | 2.50 | 2.50 | 2.50 |
| tert-butylhydroquinone | 0.014 | 0.014 | 0.014 | 0.014 | 0.014 | | 0.014 | 0.014 | 0.014 |
| Jerusalem artichoke | - | - | 144.93 | - | - | | - | - | - |
| yacon | - | - | - | 211.64 | - | | - | - | - |
| Beneo Orafti Synergy 1 | - | - | - | - | 89.83 | | - | - | - |
| sorbet with Jerusalem artichoke | - | - | - | - | - | | 470.30 | - | - |
| sorbet with yacon | - | - | - | - | - | | - | 524.11 | - |
| sorbet with Beneo Orafti Synergy1 | - | - | - | - | | - | - | - | 511.22 |
| energy value [kcal/g diet] | 4.00 | 4.02 | 3.72 | 3.71 | | 3.70 | 3.67 | 3.67 | 3.68 |

RCD - diet with recommended calcium dose – Ca content: 5000 mg/kg diet ; LCD – calcium deficient diets (60% recommended Ca dose – Ca content: 3000±0.1 mg/kg diet), i.e. LCD: low-calcium diet, LCD-JA: low-calcium diet enriched in Jerusalem artichoke, LCD-Y: low-calcium diet enriched in yacon, LCD-F: low-calcium diet enriched in Beneo Orafti Synergy 1, LCD-JAS: low-calcium diet enriched in sorbet containing Jerusalem artichoke, LCD-YS: low-calcium diet enriched in sorbet containing yacon, LCD-FS: low-calcium diet enriched in sorbet containing Beneo Orafti Synergy 1

* mineral mix in RCD group as well as vitamin mix (in all groups) were prepared according to Reeves [16]** in LCD groups corn starch was placed in mineral mix instead of calcium
