# Supplementary material for: Fructan-Enriched Diet Increases Bone Quality in Female Growing Rats at Calcium Deficiency
Source: Plant Foods Hum Nutr. 2018 May 10;73(3):172–9. doi: 10.1007/s11130-018-0671-4 (PMC6096893; doi:10.1007/s11130-018-0671-4)
Supplement: Supplementary file 4 — (DOCX 13 kb) [file 11130_2018_671_MOESM4_ESM.docx]

**Table 3** The results of two-way analysis of variance (p values)

| PARAMETER (unit) | Factor 1(kind of fructan source) | Factor 2 (form of fructan source) | Factor 1 x Factor 2 |
| --- | --- | --- | --- |
| Femoral Ca (mg/g) | 0.00 | 0.00 | 0.00 |
| Hardness (N) | 0.01 | 0.80 | 0.84 |
| DIS-ENDO_C (mm) | 0.40 | 0.38 | 0.99 |
| DIS-PERI_C (mm) | 0.52 | 0.13 | 0.76 |
| DIS-CRT_THK (mm) | 0.09 | 0.01 | 0.73 |
| DIS-CRT_A (mm^2^) | 0.09 | 0.00 | 0.67 |
| DIS-CRT_DEN (mg/mm^3^) | 0.13 | 0.02 | 0.99 |
| DIS-CRT_CNT (mg/mm) | 0.11 | 0.01 | 0.61 |
| DIS-TRAB_A (mm^2^) | 0.54 | 0.12 | 0.74 |
| DIS-TOT_A (mm^2^) | 0.53 | 0.12 | 0.75 |
| DIS-TRAB_DEN (mg/mm^3^) | 0.01 | 0.03 | 0.26 |
| DIS-TRAB_CNT (mg/mm) | 0.04 | 0.03 | 0.64 |
| DIS_TOT_DEN (mg/mm^3^) | 0.01 | 0.01 | 0.35 |
| DIS_TOT CNT (mg/mm) | 0.11 | 0.02 | 0.80 |
| MID-ENDO_C (mm) | 0.44 | 0.71 | 0.60 |
| MID-PERI_C (mm) | 0.55 | 0.59 | 0.78 |
| MID-CRT_THK (mm) | 0.75 | 0.57 | 0.42 |
| MID-CRT_A (mm^2^) | 0.66 | 0.55 | 0.71 |
| MID-CRT_DEN (mg/mm^3^) | 0.60 | 0.95 | 0.24 |
| MID-CRT_CNT (mg/mm) | 0.66 | 0.55 | 0.61 |
| MID-TRAB_A (mm^2^) | 0.53 | 0.57 | 0.78 |
| MID-TOT_A (mm^2^) | 0.49 | 0.56 | 0.75 |
| MID-TRAB_DEN (mg/mm^3^) | 0.049 | 0.82 | 0.58 |
| MID-TRAB_CNT (mg/mm) | 0.05 | 0.93 | 0.73 |
| MID-TOT_DEN (mg/mm^3^) | 0.06 | 0.73 | 0.36 |
| MID- TOT_CNT (mg/mm) | 0.57 | 0.66 | 0.70 |
| RP-CM-W (mm^3^) | 0.46 | 0.46 | 0.63 |

cortical thickness (DIS-CRT_THK, MID-CRT_THK), cortical area (DIS-CRT_A, MID-CRT_A), cortical density (DIS-CRT_DEN, MID-CRT_DEN),cortical content (DIS-CRT_CNT, MID-CRT_CNT), trabecular area (DIS-TRAB_A, MID-TRAB_A), total area (DIS-TOT_A, MID-TOT_A), trabecular density (DIS-TRAB_DEN, MID-TRAB_DEN), trabecular content (DIS-TRAB_CNT, MID-TRAB_CNT), total density (MID-TOT_DEN), total content (MID- TOT_CNT), polar strain index (RP-CM-W), as well as periosteal (DIS-PERI_C MID-PERI_C) and endosteal circumferences (DIS-ENDO_C, MID-ENDO_C)

where DIS – means distal part of bone; MID – means middle part of bone
